# Roles of airway basal stem cells in lung homeostasis and regenerative medicine

**DOI:** 10.1186/s12931-022-02042-5

**Published:** 2022-05-13

**Authors:** Meirong Wu, Xiaojing Zhang, Yijian Lin, Yiming Zeng

**Affiliations:** 1 Department of Respiratory and Critical Care Medicine, Second Affiliated Hospital of Fujian Medical University, Quanzhou, Fujian Province People’s Republic of China; 2 Stem Cell Laboratory, Second Affiliated Hospital of Fujian Medical University, Quanzhou, Fujian Province People’s Republic of China; 3 Respiratory Medicine Center of Fujian Province, Quanzhou, Fujian Province People’s Republic of China

**Keywords:** Airway basal stem cells, Lung regeneration, Homeostasis, Self-renew, Differentiation

## Abstract

Airway basal stem cells (BSCs) in the proximal airways are recognized as resident stem cells capable of self-renewing and differentiating to virtually every pseudostratified epithelium cell type under steady-state and after acute injury. In homeostasis, BSCs typically maintain a quiescent state. However, when exposed to acute injuries by either physical insults, chemical damage, or pathogen infection, the remaining BSCs increase their proliferation rate apace within the first 24 h and differentiate to restore lung homeostasis. Given the progenitor property of airway BSCs, it is attractive to research their biological characteristics and how they maintain homeostatic airway structure and respond to injury. In this review, we focus on the roles of BSCs in lung homeostasis and regeneration, detail the research progress in the characteristics of airway BSCs, the cellular and molecular signaling communications involved in BSCs-related airway repair and regeneration, and further discuss the in vitro models for airway BSC propagation and their applications in lung regenerative medicine therapy.

## Introduction

The tracheal and main stem bronchi (proximal airways) of mature human and mouse lungs are lined with a pseudostratified columnar epithelium comprising basal stem cells (BSCs) and luminal airway cells. The luminal airway cells contain secretory (or club, formerly Clara) cells, multi-ciliated cells, pulmonary neuroendocrine cells (PNECs), and goblet cells [1–7]. Together, these cells constitute the mucociliary escalator that removes inhaled particles and pathogens stuck in the mucus via mechanical actions. The airway epithelium is unceasingly exposed to insults from microbial pathogens, particles, toxicants, and mechanical trauma and serves as the first defense line. In homeostasis, airway cell proliferation is minimal with limited turnover. While upon the loss of epithelial cells, progenitor cells rapidly activate and migrate to replace lost epithelium, followed by expansion and differentiation to restore lung homeostasis. Diverse pulmonary stem/progenitor cell populations exist in distinct niches throughout the lung and mediate region-specific responses to injuries. Generally, BSCs in the proximal airways serve as resident stem cells capable of self-renewing and repopulating virtually every cell type of the pseudostratified epithelium under steady-state and after acute injury [2].

Given the progenitor property of airway BSCs, it is attractive to research their biological characteristics and how they maintain homeostatic airway structure and respond to injury. This review will elaborate on the roles of BSCs in lung homeostasis, regeneration, and disease. We will review recent advances regarding the anatomical structure similarities and divergences between humans and mice, the characteristics of airway BSCs, and the cellular and molecular signaling communications involved in BSCs-related airway repair and regeneration. We will further discuss the in vitro models for BSC expansion utilizing primary airway epithelial cells (AECs) and pluripotent stem cells (PSCs) and their applications in lung regenerative medicine therapy.

## Differences between human and mouse airways

Although the basic structure is conservative between the murine trachea and the human airways, there also exist some divergences in their anatomical structure throughout the airway [8–10]. In mice, cartilage rings are confined to the extrapulmonary trachea (diameter of ~ 1.5 mm, equivalent to the human peripheral airways). In contrast, human cartilaginous airways extend into the bronchus for several generations [9, 10]. Mucin-producing submucosal glands (SMGs), pervasive in human airways, only exist in the murine proximal trachea.

There are also interspecies differences concerning the cellular composition of the murine and human airways. Under immunohistochemical analysis, the murine bronchioles do not appear to contain Trp63 + /Krt5 + BSCs, only a tiny population of Trp63 + /Krt5 − immature basal cells presented distally [11, 12]. Thus, in mice, the pseudostratified epithelium containing Trp63-Krt5 double-positive BSCs resides exclusively in the trachea and proximal main stem bronchi [10, 12, 13]. Moreover, in the murine proximal bronchiolar airways, the pseudostratified epithelium transitions into a simple cuboidal epithelium devoid of BSCs, predominantly secretory and ciliated cells. While in humans, the pseudostratified epithelium containing Trp63-Krt5 double-positive BSCs distally down to the terminal bronchioles, and the simple cuboidal epithelium only exists in the respiratory bronchioles.

Considering the distribution of BSCs, we propose the murine trachea but not intrapulmonary airways as a better experimental platform for modeling human airways [8]. As we have seen, much of what we know about the human airway epithelium originates from studies of the mouse trachea.

## Characteristics of airway BSCs

Despite prominent differences between human and mouse airways, BSCs of the pseudostratified epithelia are molecularly and histologically analogous in both species. BSCs are morphologically simple and make intrinsic attachment to the pseudostratified epithelium basement membrane, where they are embraced by neighboring columnar cells to protect them from the luminal surface [10, 14].

**Fig. 1:**
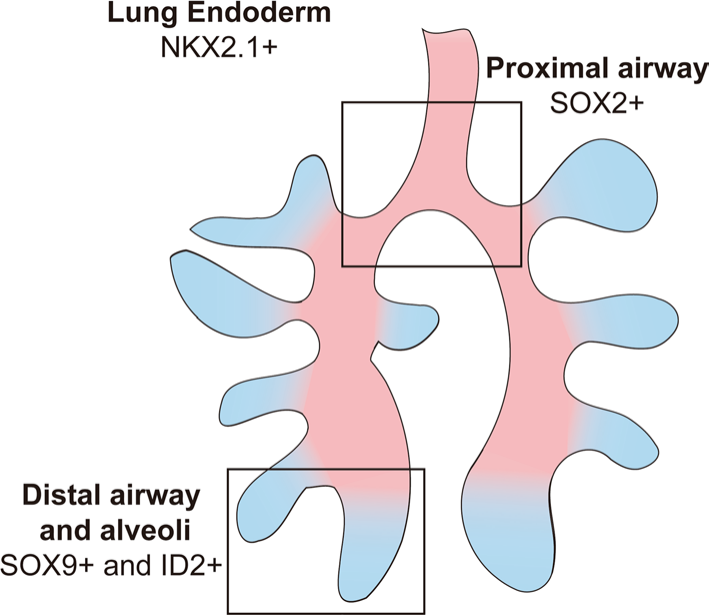
The lung endoderm. During lung development, the lung endoderm is specified from the ventral anterior foregut endoderm, and the early endoderm progenitors committed to lung epithelial lineages are marked by NK2 homeobox 1 (NKX2− 1). Following the esophagus and lung split, these endodermal progenitors (NKX2.1 +) then quickly undergo specification for either the proximal conducting airway epithelium (SOX2 +) or the peripheral distal airway epithelium (SOX9 + and ID2 +)

During lung development, the lung endoderm is specified from the ventral anterior foregut endoderm at approximately 4 to 7-week post conception in humans and embryonic day 9.0 in mice. The early endoderm progenitors on the foregut’s ventral side committed to lung epithelial lineages are marked by NK2 homeobox 1 [NKX2-1; or thyroid transcription factor-1 (TTF-1)] [15, 16], a transcription factor essential for lung formation and epithelial differentiation. Before the esophagus and trachea separate, SRY-box transcription factor (SOX) 2 is expressed at high levels in the dorsal esophageal region. Following the esophagus and lung split, SOX2 expression is established in conducting airways, whereas the peripheral region represents SOX9 and inhibitor of DNA binding (ID) 2 (Fig. 1) [17]. Recent evidence has demonstrated that Trp63 + BSCs are present early during lung development (embryonic day 10.5 in mice), and the proliferation of Trp63 + BSCs and their progenies in the adult lung largely depend on SOX2 transcriptional networks [11, 18–20].

The global transcriptome of BSCs examined by microarray analysis revealed that the BSC is a heterogeneous population. Previous lineage tracing analysis identified at least two different pre-committed BSCs subpopulations in the upper airways at steady-state [21]. One acts as a self-renewing stem cell, and the other is committed to differentiation. The existence of these pre-committed BSCs may be a feature of homeostasis and facilitate a rapid repair response. Thus, after injury, the remaining BSCs differentiate apace and acquire readily detectable club or ciliated cell markers [21]. More recently, a study performing single-cell transcriptomic analysis of fractionated epithelial cells from normal human lungs also determined four heterogeneous BSC populations that include multipotent, proliferating, secretory primed, and activated subsets [22]. The multipotent subset is defined as classical basal with a strong Wnt-signaling. The secretory primed subset shows a limited capacity for self-renewal and is primed to assume secretory cell fates. The activated subset is characterized by a stress-response signature, the activation of the p53 pathway, and contains a cell-cycle arrest signature. Through a single-cell study of brushings and biopsies from healthy human airways, Deprez et al. also described a lower Trp63 expression and higher Krt19 and Notch3 expression cell type, termed “suprabasal,” that may represent a precursor of secretory cells [23].

Different airway BSCs subpopulations express distinct and variable cell markers. The variable expressions in BSCs include the N-terminus-truncated isoform of Trp63, and cytokeratin, e.g., keratin (Krt)5 and Krt14, and the cell surface markers podoplanin (PDPN, also known as T1α), nerve growth factor receptor (NGFR), epithelial cell surface glycoprotein (Trop2), and integrin alpha chain 6 (ITGA6) [10, 24–26]. The lineage-specific cell surface markers contribute to the isolation of airway BSCs from biopsies. The signature feature of BSCs depends on their spatial location in the trachea and proximal airways and their participation in airway epithelial regeneration and repair.

## BSCs take centre stage in airway regeneration

**Fig. 2:**
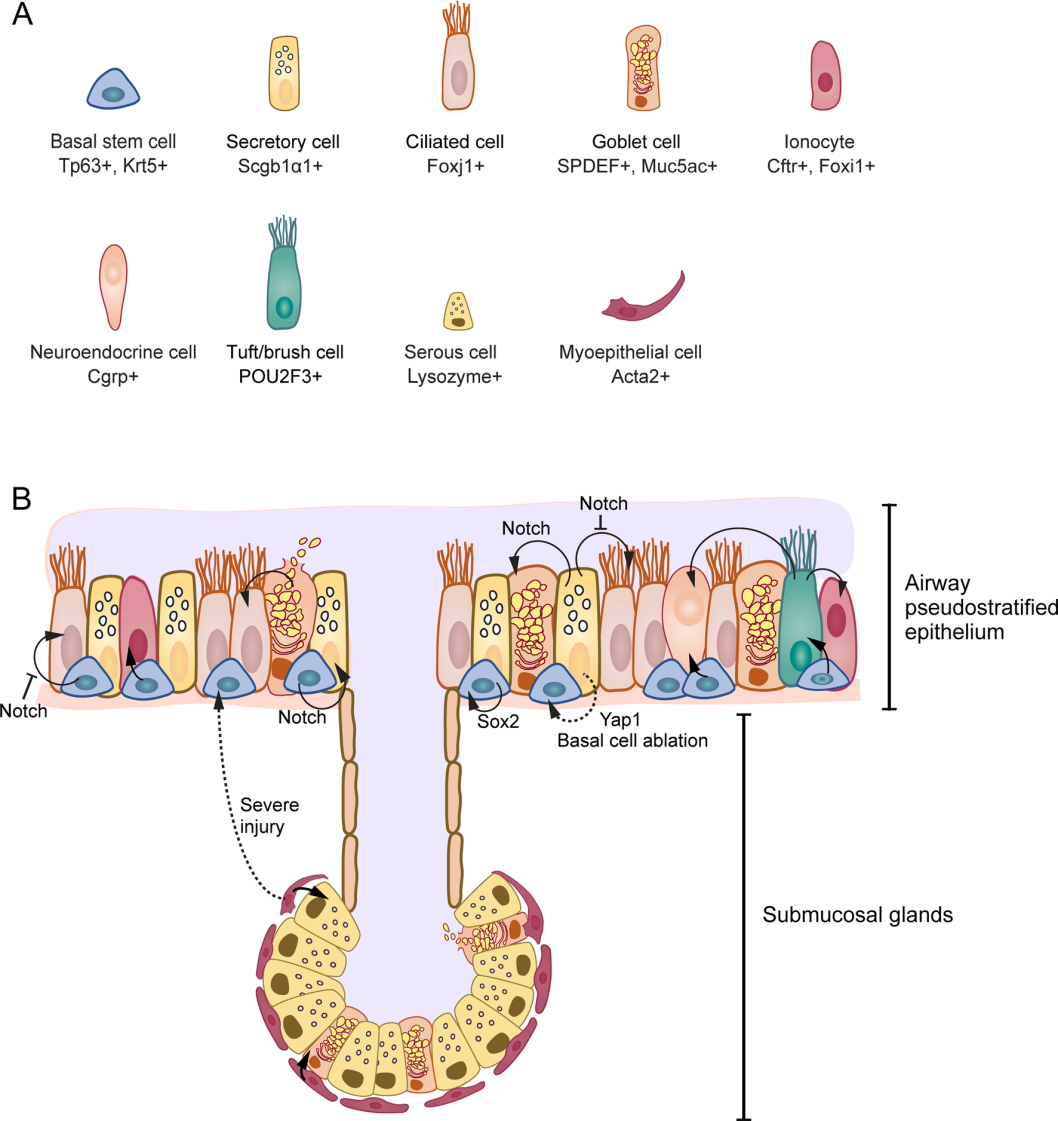
Basal Stem Cells (BSCs) Take Centre Stage in Airway Regeneration. **A** Table of cell types found in the proximal airways. **B** The conducting airway surface and submucosal glands are shown. BSCs in the airway pseudostratified epithelium function as the primary progenitors and could differentiate into virtually every airway epithelium cell population. Sox2 is required for BSC self-renewal. Notch signaling is crucial for the differentiation of BSCs and suppresses ciliated cell differentiation. In the setting of marked BSC ablation, the overexpression of Yes-associated protein 1 (Yap1) transiently stimulates secretory cell de-differentiation and partially gives rise to BSCs. Solid black arrows represent homeostatic cell fates. Dotted black arrows represent reparative strategies only activated after injury

There is little controversy that the BSCs primarily drive the proximal airway cellular renewal. BSCs function as a dedicated multipotent stem cell population capable of self-renewing and differentiating to virtually every airway epithelium cell population (Fig. 2), including luminal secretory, multi-ciliated, newly identified ionocytes, and potentially SMG epithelial cells during postnatal growth, homeostasis, and epithelial repair following the loss of luminal cells [1–6, 27]. Numerous studies using lineage tracing analysis and single-cell transcriptomic work also exhibited that relatively rare epithelial cell subsets such as PNECs and tuft/brush cells might descend from BSCs [28–32]. In homeostasis, BSCs typically maintain a quiescent state. However, when exposed to acute injuries by either physical insults, chemical damage, or pathogen infection, the remaining BSCs increase their proliferation rate apace within the first 24 h and differentiate into multi-ciliated and secretory cells [33].

### Secretory cells

In addition to the BSCs, the secretoglobin family 1A, member 1 (Scgb1α1) positive secretory cells, which are descendants of BSCs, also act as a facultative progenitor population. In the steady-state, secretory cells can replace their senescent population and generate multi-ciliated cells, specifically in the mouse’s bronchioles, where BSCs are not found [34–36]. Intriguingly, the committed, mature tracheal secretory cells displayed plasticity to self-renew, de-differentiate, and contribute to the bona fide BSC pool under extreme physiologic stresses in the setting of marked BSC ablation (Fig. 2) [35]. Through grafting luminal secretory cell populations onto denuded trachea in mice, followed by implanting the reseeded tracheal xenografts into immunodeficient mice, Liu and colleagues demonstrated that luminal secretory cells could regenerate the tracheal epithelium [37]. This study showed that tracheal secretory cells could give rise to BSCs after injury. Subsequent lineage tracing analysis also showed that a tiny proportion of labeled BSCs were derived from lineage-labeled secretory cells following sulfur dioxide (SO_2_)-induced airway injury [34]. Additional experiment using diphtheria toxin to precisely ablate endogenous Krt5 + BSCs has further confirmed that secretory cells could proliferate rapidly to compensate for BSC loss. Around 8% of these cells lost secretory cell markers while acquiring BSC markers. These de-differentiated BSCs were molecularly and morphologically indistinguishable from original native BSCs, both of which could persist and participate in the long-term maintenance of the epithelium and injury repair [35]. However, this secretory cell plasticity was suppressed when more than 20% of the BSCs remained intact, suggesting a tightly restrained fail-safe reparative strategy [34]. Concerning the potential mechanisms underlying this de-differentiation, Zhao and colleagues have discovered that the overexpression of Yes-associated protein 1 (Yap1) in secretory cells transiently stimulates de-differentiation and partially generates BSCs with characteristic pyramidal morphology, and the BSC-specific marker T1α and Trp63 [38]. This partially reprogramming event requires the persistent expression of the Yap1 transgene. However, the precise mechanisms underlying de-differentiation remain unclear and require further investigation.

### Ciliated cell differentiation

Ciliated cells, which play a critical part in mucociliary clearance, are terminally differentiated cells and do not have the differentiation potential either in the steady-state or under injury. Both BSCs and secretory cells could differentiate into ciliated cells [17, 21, 39–41]. Analysis of pseudo temporally ordered transcriptome trajectories indicates that the homeostatic production of ciliated cells by BSCs is transitioned through a secretory cell state. This conversion of secretory cells into ciliated cells is called trans-differentiation [39, 41]. Nevertheless, during injury repair, it appears that BSCs directly yield ciliated cells without passing through a secretory cell fate [21, 39]. Following the SO_2_ ablation of luminal epithelial cells, Pardo-Saganta and colleagues conferred that BSCs segregate into two discrete subpopulations: the precursors of secretory cells exhibit active Notch2 intracellular domain (N2ICD), and the precursors of ciliated cells that express c-myb [39]. The mechanisms that govern this injury-induced separation remain obscure. Through single-cell transcriptomic analysis, García and colleagues recently described that goblet cells can also act as precursors of ciliated cells in an in vitro cell culture differentiation model and fresh biopsies from human homeostatic bronchi and newborn pig trachea [32]. They also demonstrated that deuterosomal cells, which are involved in the early steps of multiciliogenesis, could be defined as a precursor subgroup of multi-ciliated cells [32].

### Ionocytes

Using single-cell RNA-sequencing and in vivo lineage tracing experiments, a pair of recent studies identified a novel rare airway cell population, the pulmonary ionocytes, which account for nearly 1% of airway epithelial cells [29, 42]. The pulmonary ionocytes express Forkhead box I1 (FoxI1), multiple subunits of the V-ATPase (Atp6v1c2 and Atp6v0d2), Cochlin, ASCL3, and the cystic fibrosis (CF) transmembrane conductance regulator (CFTR). The functional defect of the CFTR protein in ionocytes plays a critical contributor to the monogenic, recessive disease CF. Previous studies revealed that the ionocytes are decedents frequently and directly originating from BSCs [29, 42]. Recently, accompanying knockout studies, Goldfarbmuren et al. demonstrated that tuft-like cells are the likely progenitor of PNECs and ionocytes [43]. Their identity and functional dedications to normal lung physiology and CF remain elusive.

### Glandular myoepithelial cells

In addition to the pseudostratified surface epithelium mentioned above, the airway epithelium consists of the SMGs, which control the secretion of proteins and mucus crucial in airway innate immunity. BSCs in the ducts of SMGs and Krt-5 expressing glandular myoepithelial cells (MECs) can serve as multipotent progenitors from which serous, mucus, and ductal cell types are produced [44–46]. Through lineage tracing techniques, two recent reports have shown that glandular MECs express Alpha-actin-2 (Acta2) and an inducible Acta2 promoter-driven creERT2 mouse line can be used to trace their destiny following injury [45, 46]. These investigators found that tracheal SMGs might serve as a protected stem cell niche upon severe injury. A portion of Acta2 + MECs could proliferate, migrate into the airways, and repopulate the damaged pseudostratified surface epithelium after severe but not moderate injury or homeostatic turnover. Thus, they defined glandular MECs as reserve multipotent stem cells of the surface airway epithelium and dedicated SMGs stem cells. These glandular MECs are Wnt responsive and have a great capacity to regenerate the airway epithelium [47].

## Signaling pathways involved in BSCs related airway repair and regeneration

**Fig. 3:**
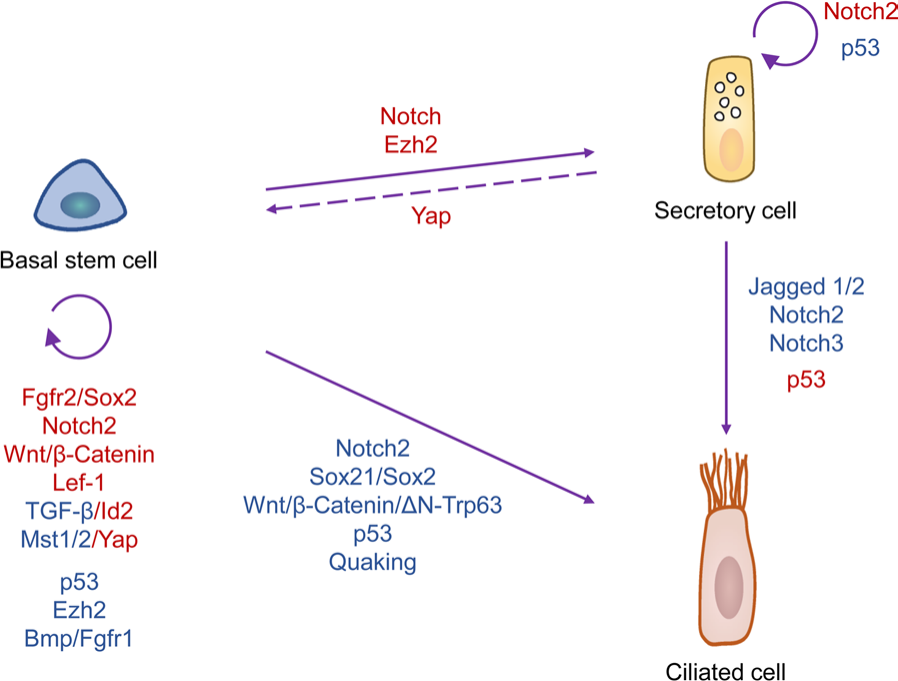
Related Molecular Mechanisms that Regulate Self-renew and Differentiation properties of BSCs. Solid purple arrows represent homeostatic cell fates. Dotted purple arrows represent reparative strategies only activated after injury. The red text next to the purple arrow represents the corresponding molecular mechanism facilitating the process. The blue text next to the purple arrow represents the corresponding molecular mechanisms that repress the process

Numerous studies are ongoing to uncover the molecular mechanisms that regulate the progenitor property of BSCs (Fig. 3). Fibroblast growth factor receptor (Fgfr2)-mediated transcription of Sox2 is required for BSC self-renewal [20], while BSCs differentiation is strongly affected by the level of Notch signaling [22, 30, 39, 40, 48–53]. The Notch signaling plays diverse and pivotal roles in BSC lineage commitment and differentiation regulation during lung morphogenesis and airway repair. Notch preferentially provokes acquiring a secretory cell fate over a multi-ciliated and neuroendocrine cell fate [40]. Consequently, loss of Notch signaling makes for shunting of BSCs into the multi-ciliated destiny. Repression of the Notch ligands jagged 1 and jagged 2 can also lead to trans-differentiation of secretory cells into multi-ciliated cells [30, 39, 41]. Pardo-Saganta et al. previously demonstrated that BSCs provide a Notch2 signal to their secretory cell progenies to maintain their secretory cell state and inhibit trans-differentiation [39]. This type of forwarding signal demonstrates a direct connection between BSC and its progenies, which could help preserve the pseudostratified architecture of the upper airway. Recently, Carraro and colleagues further confirmed that Notch2 maintains undifferentiated BSCs and restricts basal-to-ciliated differentiation, and Notch3 functions to restrain secretory differentiation [22]. Moreover, ectopic Notch activation in the developing and postnatal lung can increase the goblet cell number, defined as mucous metaplasia [40, 54]. The ability of Notch to regulate BSC stem/progenitor function and cell fate decisions may offer a practical approach to promoting airway epithelial regeneration after acute lung injury.

Apart from Notch signaling, the proliferation and differentiation of BSCs are regulated by multiple highly conserved signaling inputs. For example, Sox21 and Sox2 are co-expressed and involved in balancing BSCs differentiation to ciliated cells, that Sox21 can act as a suppressor of differentiation when Sox2 expression levels are high and when BSCs are prone to differentiate55. Wnt/β-Catenin prevents differentiation in BSCs of ciliated cells by activating ΔN-Trp63, and prolonged overactivation of Wnt signaling could lead to BSC hyperplasia [56]. Furthermore, Lef-1 in canonical Wnt signaling is required for BSCs to maintain a multipotent state. Loss of Lef-1 significantly reduces the regenerative capability of BSC following injury [57]. The inactivation of p53 promotes BSCs self-renewal in vitro [58], and direct inhibition of the MDM2/p53 interaction, which increases the p53 expression level, antagonizes the differentiation process in BSCs [59]. Mature BSCs also maintain moderate Id2 expression to sustain proliferative potential, and TGF-β signaling regulates tissue regeneration through recurrent Id2 activation following injury [60]. Inactivation of the Polycomb repressor complex gene Ezh2 induces BSC proliferation and reduces secretory cell differentiation in the proximal airway epithelium [18, 19]. Loss of the Hippo kinases, Mst1 and Mst2, increases nuclear Yap, enhances airway epithelial proliferation, maintains a steady-state BSC identity, and prevents cell differentiation of human bronchial epithelial cells in vitro [38]. Moreover, overexpression of Yap in adult tracheal BSCs leads to BSC hyperplasia and stratification [61]. Bmp and Fgfr1 signaling inhibit BSC proliferation under steady-state and following tracheal injury [62, 63], while levels of Bmp transcription decrease to permit expansion, therefore, facilitating repair [63]. Additionally, the knockdown of RNA-binding protein Quaking promotes the differentiation of BSCs into ciliated cells by inducing IL-6 mRNA expression [64].

The epithelial-mesenchymal cross-talks are also essential for establishing a functional BSC niche in the lung airway. Using kidney capsule engraftments and culture of lung explants, Ruiz and colleagues found that p38α induces the expression of SDF-1 in airway epithelium BSCs to recruit and activate stromal cells, which secrete TNFα and in turn activate the BSCs, establishing a positive feedback loop [65]. Additionally, the BSCs in the proximity of stromal cells maintain undifferentiation and remain self-renewal properties. The sonic hedgehog (Shh) signal secreted by the adult lung epithelium at homeostasis actively suppresses adjacent mesenchyme proliferation [66]. Epithelial-specific deletion of Shh relieves the mesenchymal quiescence and subsequently forms feedback to increase epithelial cell proliferation and differentiation [66, 67]. Trachea BSCs during homeostasis and differentiated airway epithelial cells upon injury form a Hippo-Yap-mediated localized Fgf10 expressing stromal niche, which maintains the stem cell state of BSCs and induces the BSC population to spread to non-cartilaginous airways, thus facilitating regeneration [11, 68]. Merlin degradation and downregulation of Hippo signaling cause an increase in Yap-mediated epithelial Wnt7b expression, which acts in a paracrine pattern to promote Fgf10 expression in the adjacent airway smooth muscle cells [68–70]. This airway smooth muscle cells-derived Fgf10 maintains and amplifies the BSC population via Fgf10-Fgfr2b signaling [71]. Furthermore, BSCs could recruit immune cells, such as CD4 + Th2 cells, innate lymphoid cells, and NKT cells, to the airways via releasing interleukin-33 (IL-33) [72]. Hence, they may also play an essential immunomodulatory function.

## Application of BSCs in lung regeneration

### Anomalous BSC behavior in respiratory diseases

Serving as stem/progenitor cells for lung regeneration, BSCs are dynamically regulated in disease. Pathological airway remodeling via BSCs behavior modifications, including BSC hyperplasia, squamous metaplasia, goblet cell metaplasia, and goblet cell hyperplasia, are involved in the pathogenesis of several progressive respiratory diseases such as chronic obstructive pulmonary disease (COPD), CF, asthma, idiopathic pulmonary fibrosis (IPF), and lung squamous cell carcinoma (LUSC) [22, 41, 73–82].

The detection of BSCs within bronchoalveolar lavage fluid of IPF patients is often related to poor prognosis [79], and BSCs are specifically biased toward the expansion of the secretory primed subset in IPF [22]. Thus, BSC hyperplasia and associated mucosecretory dysfunction are involved in the development of IPF. BSC hyperplasia combined with goblet cell hyperplasia is often observed in the COPD samples [73, 78], and BSCs from patients with COPD revealed a significant upregulation of genes associated with stress and stress and other damaging factors [78]. Previous histological reports indicated goblet cell hyperplasia and BSC hyperplasia in the CF airways [80]. Nonetheless, a recent study performing a single-cell transcriptomic analysis of CF airways revealed a reduction in proliferating BSCs [81]. Goblet cell metaplasia is observed after sensitization to the allergen in asthmatic airways [41]. Moreover, the resemblance of BSCs to a squamous cell carcinoma phenotype has supported that BSCs may be a leading candidate cell-of-origin for lung squamous cell carcinoma (LUSC), which occurs principally in the central airways [76, 77, 82]. BSC hyperplasia, squamous metaplasia, and different severities of bronchial dysplasia are often observed in LUSC, inducing a stepwise progression from precancerous pathologies to carcinoma in situ and further invasive and metastatic LUSC [82].

### in vitro models for BSC expansion utilizing primary airway epithelial cells

The stem cell properties make BSCs a desirable ex vivo producing cell type for modeling airway diseases and a leading contender for cell-based regenerative therapies to reconstruct the airway epithelium. The in vitro culture models have been simple and powerful tools for exploring BSC biology. However, to date, approaches that allow BSC extensively in vitro serial propagation and sustain bona fide self-renew and differentiation potential are still waiting to establish. Various BSC culture strategies, such as immortalization, improved two-dimensional (2D) cell culture conditions [83], and enhanced three-dimensional (3D) culture conditions [24, 35, 83–86], have been built to extend the primary BSC culture lifetime.

The induction of immortalization factors generally could construct primary cells into immortalized BSC lines. For example, the immortalized BSC lines—BEAS-2B and 16HBE14o—were created by introducing simian virus 40 (SV40) large T antigen to primary AECs [83, 87]. Nevertheless, the BEAS-2B cells were unable to form tight junctions. Besides, since the SV40 large T antigen functionally suppresses the tumor suppressor genes p53 and Rb, the 16HBE14o cells turn tumorigenic over serial passaging [88]. Additionally, the induction of CDK4 and hTERT also immortalize the HBEC-KT and HSAEC1-KT cell lines, which preserve a stable phenotype over serial passaging and differentiate into both ciliated and goblet cells in the air–liquid interface (ALI) or 3D culture [89–92]. However, it is uncertain how well these immortalized cell lines sustain the physiology of normal airway BSCs, which constraints their utilities in generating complex in vitro lung models.

Primary AECs freshly isolated from lung tissue consist of multiple cell types, but BSCs are the predominantly expanding population under in vitro submerged culture. The ALI cultures of primary AECs are a well-established in vitro 2D model traditionally used to probe BSC biology. The ALI models encourage primary AECs to differentiate into a pseudostratified epithelial layer comprising basal, ciliated, and mucus-producing secretory cells [85, 93, 94]. This technique offers an instrumental platform to explore the dynamic repair processes following injury, the cellular mechanisms of airway epithelium damage and even drug discovery to treat specific diseases. Nevertheless, this is a time-consuming approach, and airway BSCs rapidly miss their multipotent differentiation potential and experience premature deterioration after only a few passages in this culture condition, which hampers their use in regenerative medicine.

The BSC-based tracheal tissue-engineered graft is a promising therapeutic alternative for untreatable airway diseases, such as congenital tracheal stenosis and upper airway tumors. It represents essential to develop a method to expand and differentiate large numbers of airway BSCs for bioengineering applications. Janes and colleagues recently described a procedure that airway BSCs and mitotically-inactivated mouse embryonic fibroblast feeder cells coculture in a medium containing the Rho-associated kinase (ROCK) inhibitor Y-27632 [84, 86]. This culture technique efficiently induces successful ex vivo long-term population doublings of airway functional BSCs. These cells retain tissue-appropriate differentiation capacity, persist in karyotype stability, express markers typical of airway BSCs, and do not express characteristic markers of human pluripotent stem cells (hPSCs). This culture method offers a novel platform for more realistic in vitro airway models, high-throughput, high-content translational lung research, human tissue-engineered tracheal transplants, and clinical cellular therapy. Janes and colleagues found a robust hepatocyte growth factor (HGF) production in this culture system but could not find that the HGF signaling promotes BSC expansion [95]. However, they found that HGF did induce phosphorylation of GAB2 and STAT6, targets of HGF receptor MET, which indicates that some more complex cross-species protein interactions may exist in this culture system [95]. Besides, the addition of xenogeneic feeder cells challenges meeting supervisory requirements for cell therapy product manufacture. Therefore, a well-defined feeder-free culture system is preferred and expected to increase the culture system consistency.

Through analysis of signaling pathways, the Rajagopal group observed that the TGFβ/BMP/SMAD pathway is intensely active in luminal epithelial cells but repressed in BSCs [96]. Thus, they exploit a dual SMAD inhibition culture system in the absence of feeder cells to extend human and murine BSC in vitro culture longevity, and cells in this culture system could sustain the ability to differentiate into functional tissues [96]. Likewise, the TGF-β pathway inhibition by A83-01 combined with ROCK inhibition and isoproterenol supplement also could support long-term BSC expansion in the absence of feeder cells. However, their physiologic properties progressively change over long-term in vitro expansion, demonstrating that more future work will be needed to resolve this limitation.

To better simulate the bona fide complex lung structural micro-environment and eventually manufacture a brand-new lung for patients with end-stage lung disease, researchers have made great efforts in the culture of 3D organoids and tissue-engineered lungs. The 3D culture of “tracheospheres” or “bronchospheres” organoids, which contain basal and luminal cell populations that reveal the lung structure, has been established by implanting the primary AECs within the extracellular matrix (ECM) substrates [24, 40, 86, 96]. These spherical organoid colonies are notably suitable for studying the proliferative and stem cell properties of BSCs, investigating the epithelial response to specific stimuli, and recognizing various instructive signals that influence BSC differentiation. Researchers have recently developed a tissue-engineered lung that reseeds human lung cells onto the decellularized rodent or human biological or bioengineered synthetic scaffolds [97]. This tissue-engineered lung serves as an exceptional tool for exploring the effect of ECM on BSC differentiation and repair.

### Airway BSCs derived from directed differentiation of PSCs

Additionally, directed differentiation of hPSCs into airway BSCs represents a promising implementation that overwhelms the hurdles of tissue accessibility, donor heterogeneity, and tissue quality and would have broad implications in regenerative medicine. Directed differentiation is an experimental paradigm that in vitro stepwise imitates the signaling events that control cell fate determinations in the embryo. Several groups have triumphantly derived proximal airway epithelium, involving airway BSCs, from human or mouse PSCs [98–105]. Mou et al. have firstly reported the generation of multipotent Nkx2.1 + immature lung progenitor cells from mouse embryonic stem cells and CF patient-specific induced pluripotent stem cells (iPSCs) [102], which could mature into Nkx2.1 + /Sox2 + proximal progenitor cells and Nkx2.1 + /Trp63 + airway BSCs in vitro and differentiate into airway epithelium. Furthermore, Konishi and colleagues recently have effectively engendered the hPSC-derived bronchospheres in a 3D ECM [100]. Supplementation of a Notch pathway inhibitor, DAPT, could induce these spheroids to generate PNECs, mucus-producing goblet cells, functional multi-ciliated cells, and without alveolar epithelial cells. More recently, using a dual fluorescent reporter system, Hawkins et al. demonstrated the successful generation of putative NKX2.1^GFP+/^Trp63^tdTomato+^ BSCs, which share an essential characteristic of their endogenous counterparts, from human iPSCs [104, 105]. Although most studies differentiating hPSCs into airway progenitor cells utilize analogous experimental strategies, slight differences in methods persist. No consensus has been achieved on the standardized directed differentiation protocol. Therefore, further studies are needed to obtain hPSCs-derived airway BSCs with corresponding physiological characteristics to their in vivo counterparts.

### Airway BSC-based regenerative medicine therapy

The aptitude of pulmonary resident stem/progenitor cell populations to restore lung homeostasis and the success and promise of adoptive cell therapy in immune disorders’ treatment inspire researchers to probe into the feasibility of epithelial stem/progenitor cell transplantation in lung regenerative therapy. Currently, several researchers have substantiated the successful transplantation or engraftment of airway BSCs derived from both mice and humans in injured mouse lungs. Above mentioned in vitro models of BSCs or BSC-based tracheal tissue-engineered grafts derived from primary AECs or PSCs pave a pathway for cell-based airway regenerative therapies. Recently, manipulating the in vitro airway BSCs model using other technologies, especially gene-editing, has also been an attractive therapeutic option to safely and effectively correct the CFTR mutations in CF airways [106]. Zuo and colleagues have performed two exploratory clinical trials to treat COPD using autologous bronchial BSCs (NCT03188627 and NCT03021681). They observed some alleviations of symptoms and pulmonary function enhancement in patients receiving cell transplantation [107]. Airway BSC-based regenerative therapy displays a striking prospect.

## Discussion and future prospects

The progenitor property of airway BSCs indicates its potential in pulmonary tissue regenerative medicine and attracts many researchers to work in this field. Nonetheless, there are still some problems to be solved. Most researchers employ the murine trachea as the experimental platform, and there is a remarkable absence of valuable research in large animals that more closely imitate the human pulmonary pathophysiology, such as domestic rabbit and porcine, et al. [108, 109]. Although airway BSCs are recognized resident stem cells, the detailed mechanisms of their self-renew and differentiation and how they took part in the epithelial-mesenchymal cross-talk are intricate and still awaiting further study. Previous research exploring airway BSC biology and its role in lung development is mainly based on employing severe injuries combined with in vivo lineage-tracing studies. However, observations in animal models under extreme stresses do not necessarily entirely reflect physiological tissue turnover in humans, and specific cell genetic labeling may not necessarily comprehensively provide a panorama of the airway epithelial cell hierarchies [32]. Single-cell RNA-sequencing has emerged as an attainable approach for lineage inference combined with pseudo temporal ordering and computational tools such as Monocle and RNA velocity [110]. On this ground, future research may investigate cell diversity and lineage hierarchies more from fresh human airway tissues or in vitro cell culture differentiation model of human airway epithelium, thus delineating the atlas of the human airway.

As a chief contender for cell-based regenerative therapy in airway diseases, the ex vivo expansion culture system to sustain the multipotent property of airway BSCs has not been reached, which may delay the progress of their application in clinical practice. Therefore, a stabilized culture strategy that allows expansively in vitro BSCs serial propagation should be established. Further excavation is needed to obtain enough delivered viable cells and investigate the transplanted cells’ protective mechanism, thus accelerating the translation of experimental BSC cell therapy to clinics. Human BSC transplantation clinical trials are still very early and exploratory. Thus, more pre-clinical successes are required for validation in quality control, transplantation efficiency, and transplantation efficacy. Nevertheless, it is worth looking forward to airway BSC-based regenerative medicine therapy becoming a part of routine frontline therapy in airway diseases in the future.

## Data Availability

Not applicable.

## Abbreviations

Acta2: Alpha-actin-2
AECs: Airway epithelial cells
ALI: Air–liquid interface
BSCs: Basal stem cells
CF: Cystic fibrosis
CFTR: Cystic fibrosis transmembrane conductance regulator
COPD: Chronic obstructive pulmonary disease
ECM: Extracellular matrix
Fgfr2: Fibroblast growth factor receptor
FoxI1: Forkhead box I1
HGF: Hepatocyte growth factor
hPSCs: Human pluripotent stem cells
ID: Inhibitor of DNA binding
IL-33: Interleukin-33
IPF: Idiopathic pulmonary fibrosis
ITGA6: Integrin alpha chain 6
Krt: Keratin
LUSC: Lung squamous cell carcinoma
MECs: Myoepithelial cells
NGFR: Nerve growth factor receptor
NKX2-1: NK2 homeobox 1
N2ICD: Notch2 intracellular domain
PDPN: Podoplanin
PNECs: Pulmonary neuroendocrine cells
PSCs: Pluripotent stem cells
ROCK: Rho-associated kinase
Scgb1α1: The secretoglobin family 1A, member 1
Shh: Sonic hedgehog
SMGs: Submucosal glands
SO_2_: Sulphur dioxide
SOX: SRY-box transcription factor
SV40: Simian virus 40
TTF-1: Thyroid transcription factor-1
Yap1: Yes-associated protein 1
2D: Two-dimensional
3D: Three-dimensional
